# Recurrence of Postoperative Stress-Induced Cardiomyopathy Resulting from Status Epilepticus

**DOI:** 10.1155/2017/8063837

**Published:** 2017-01-22

**Authors:** Grant A. Miller, Yousef M. Ahmed, Nicki S. Tarant

**Affiliations:** Department of Critical Care Medicine, Naval Medical Center Portsmouth, Portsmouth, VA, USA

## Abstract

*Introduction*. Classically, stress-induced cardiomyopathy (SIC), also known as takotsubo cardiomyopathy, displays the pathognomonic feature of reversible left ventricular apical ballooning without coronary artery stenosis following stressful event(s). Temporary reduction in ejection fraction (EF) resolves spontaneously. Variants of SIC exhibiting mid-ventricular regional wall motion abnormalities have been identified. Recent case series present SIC as a finding in association with sudden unexplained death in epilepsy (SUDEP). This case presents a patient who develops recurrence of nonapical cardiomyopathy secondary to status epilepticus.* Case Report*. Involving a postoperative, postmenopausal woman having two distinct episodes of status epilepticus (SE) preceding two incidents of SIC. Preoperative transthoracic echocardiogram (TTE) confirms the patient's baseline EF of 60% prior to the second event. Postoperatively, SE occurs, and the initial electrocardiogram exhibits T-wave inversions with subsequent elevation of troponin I. Postoperative TTE shows an EF of 30% with mid-ventricular wall akinesia restoring baseline EF rapidly.* Conclusion*. This case identifies the need to understand SIC and its diagnostic criteria, especially when cardiac catheterization is neither indicated nor available. Sudden cardiac death should be considered as a possible complication of refractory status epilepticus. The pathophysiology in SUDEP is currently unknown; yet a correlation between SUDEP and SIC is hypothesized to exist.

## 1. Introduction

Classically, stress-induced cardiomyopathy (SIC), more commonly known as takotsubo cardiomyopathy, displays the pathognomonic feature of echocardiographic, reversible left ventricular apical ballooning without angiographic coronary artery stenosis occurring after a stressful event [1, 2]. SIC is much more common among females than males, especially in older adults [3]. Typically, the temporary decline in ejection fraction (EF) resolves within a few days to weeks. More recently, variants of SIC that exhibit mid-ventricular regional wall motion abnormalities have been identified [3, 4]. Recent data suggests that SIC may be secondary to status epilepticus (SE) in up to 56% of cases [5]. Additionally, recurrence is associated with sudden unexplained death in epilepsy (SUDEP) in 3% of cases [5]. It is important to note that a study by Belcour et al. reports incidence of SIC in ICU patients admitted with convulsive status epilepticus, but these refractory cases do not represent all cases [5]. Recent case series present SUDEP as a one-time finding in a possible association with neurologic pathologies (i.e., seizures) [6]. In this case, we are presenting a patient who develops recurrence of nonapical SIC secondary to status epilepticus.

## 2. Case Presentation

**Figure 1:**
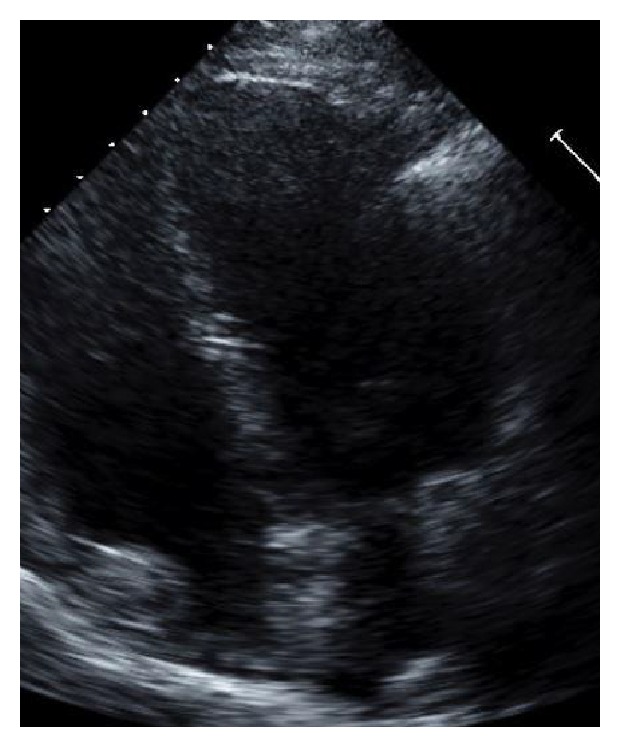
Preoperative TTE, end diastole (preop pics).

**Figure 2:**
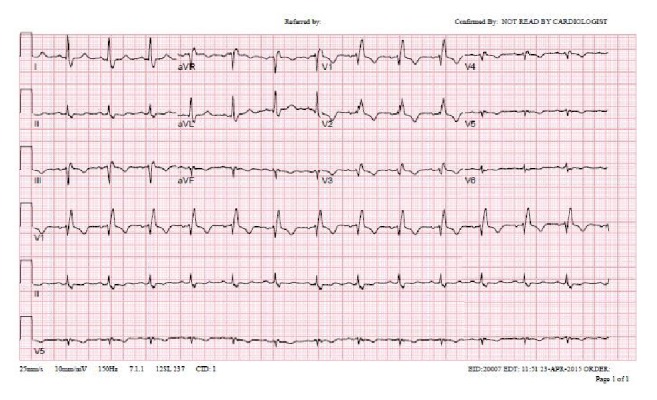
Preoperative/4 months after prior takotsubo cardiomyopathy (preop pics).

Our patient is a 49-year-old female with medical history significant for hypertension, refractory status epilepticus, and recent diagnosis of endometrioid endometrial carcinoma initially admitted to our institution for elective total abdominal hysterectomy and bilateral salpingooophorectomy. She required an ICU admission for postoperative generalized tonic-clonic status epilepticus. Of note, she was hospitalized six months priorly for new onset seizure and subsequently identified stress-induced cardiomyopathy. During her previous hospitalization, her SIC resolved 10 days after initial identification of mid-ventricular to apical akinesia, demonstrated by EF of 25% on echocardiogram and minimal increase in troponin T to 0.03 (reference < 0.01). A coronary angiogram did not occur as part of the work-up of cardiomyopathy. Resolution of her episode of SIC was defined by a repeat echocardiogram showing EF of 60% without identifiable wall motion abnormality (Figure 1, preoperative TTE, end diastole). After the first hospitalization, she had a follow-up nuclear stress test with EF of 72% without wall motion abnormality and electrocardiogram (ECG) with normal sinus rhythm (NSR) and right bundle branch block (RBBB) (Figure 2, preoperative/4 months after initial SIC).

**Figure 3:**
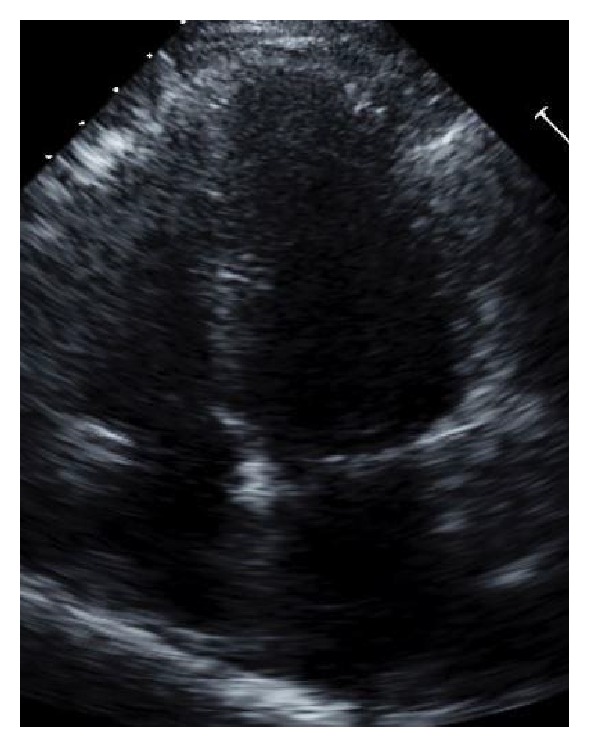
Preoperative TTE, end systole (preop pics).

**Figure 4:**
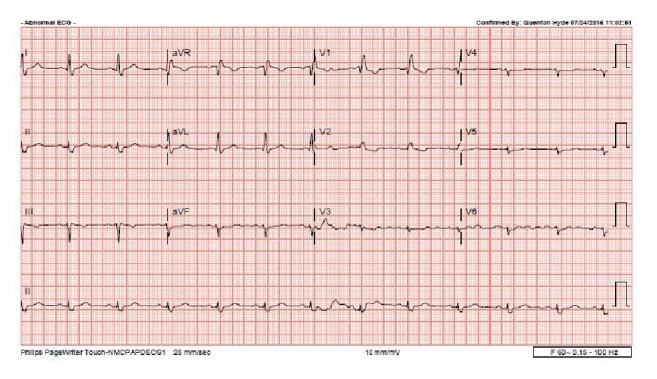
Immediately after seizure (postop pics).

**Figure 5:**
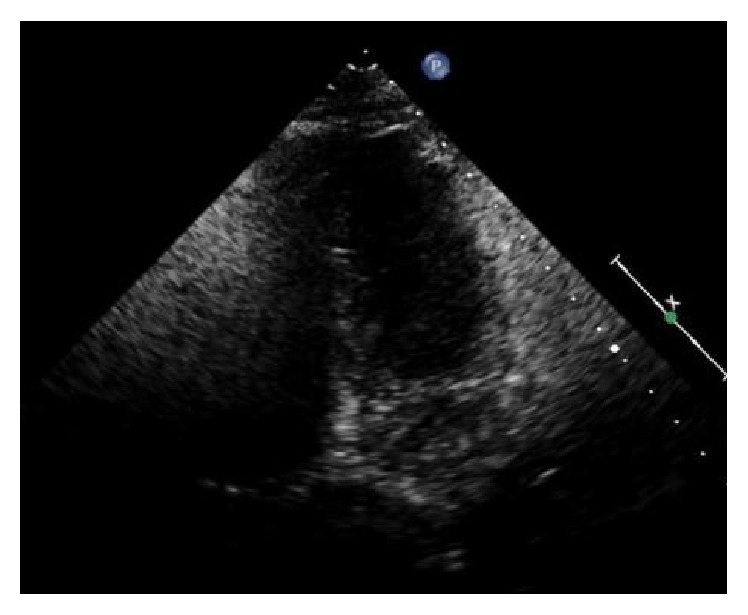
Mid-ventricular akinesis, end systole (postop pics).

**Figure 6:**
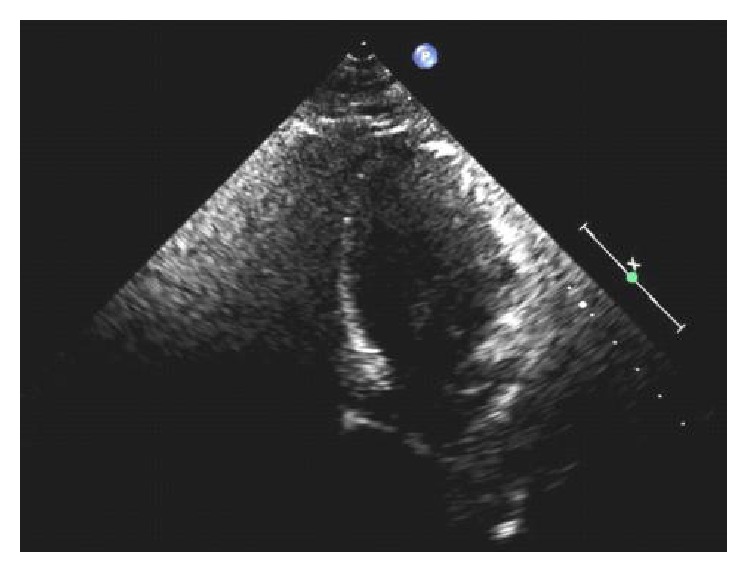
Mid-ventricular akinesis, end diastole (postop pics).

Six months following her initial hospital discharge, a preoperative TTE demonstrated an EF of 60–65% without wall motion abnormality and an ECG having NSR and RBBB (Figures 1–3). In the postanesthesia care unit (PACU), though initially stable after surgery, the patient experienced a seizure and developed refractory status epilepticus (RSE) requiring admission to the ICU. Her seizures were described as being generalized tonic-clonic. Following management of her episode of RSE, her ECG demonstrated no ST segment elevation and sinus tachycardia (Figure 4, immediately after seizure). During this period, she was transferred to the ICU and a bedside TTE was performed. Though quality of imaging was limited by tachycardia, inferior and lateral wall akinesia was identified. Troponin I was obtained and elevated at 0.563 initially and after 6 hours at 0.762 (reference < 0.01). A formal TTE was performed the following day, which revealed posterior wall akinesia, septal wall dyskinesia, and an EF of 15–20% (Figures 5 and 6, mid-ventricular akinesis at end systole and end diastole). Coronary angiogram was initially not performed due to the risk-benefit analysis of continued seizure activity on triple antiepileptic therapy and breakthrough benzodiazepine therapy. Combination of previous history of SIC after seizure, quick resolution of cardiac complications, and conversion to benzodiazepine coma to stop all seizure activity precluded the ability to perform coronary angiogram.

**Figure 7:**
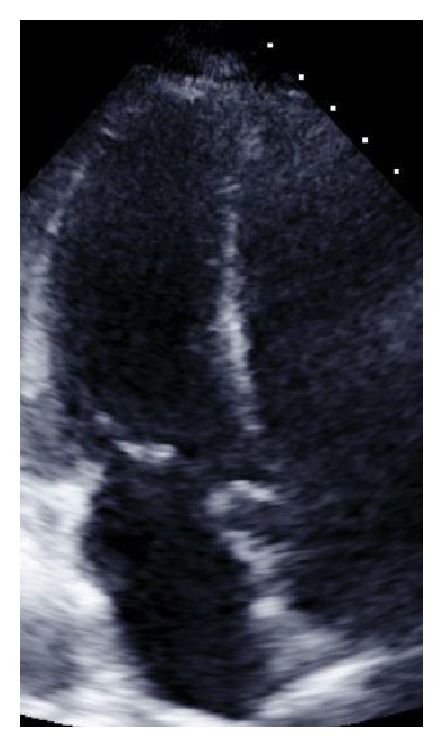
Resolution of SIC, end systole** (**SIC resolution pics).

**Figure 8:**
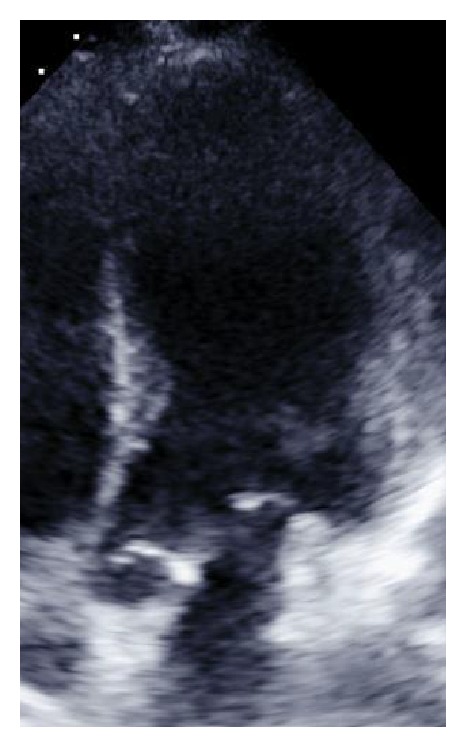
Resolution of SIC, end diastole (SIC resolution pics).

Four days after the initial cardiac event, another formal TTE was obtained which demonstrated no wall motion abnormality and her EF returned to a baseline of 60%, which was consistent with her previous admission (Figures 7 and 8, resolution SIC, end systole and end diastole). Three weeks postoperatively, she was hypertensive and tachycardic after extubation and required labetalol to control catecholamine surge to prevent exacerbation of SIC. Prior to discharge, she was restarted on carvedilol to prevent recurrent cardiomyopathy, which was prescribed after her previous event. The patients' cardiovascular findings continued to improve after resolution of cardiomyopathy.

## 3. Discussion

During the management of our patient, close evaluation of ECG changes became necessary due to prolonged QT interval (QTI) effects of antiepileptic medications. Consideration for SIC after seizure warrants checking troponins as undiagnosed SIC may progress to cardiogenic shock and SUDEP [6]. As previously described, SIC may be secondary to surgery and the status may be secondary to cerebral hypoxia or metabolic abnormalities in the setting of surgery [7]. A cardiac catheterization could not be performed due to risks associated with RSE; in addition, other diagnostic information indicates SIC as a likely diagnosis. However, cardiac catheterization is not mandatory in typical SIC. Resolution of cardiac dysfunction ultimately occurred within four days, excluding the need for cardiac catheterization. The decision not to catheterize the coronary arteries was made on clinical risk-benefit analysis of patient safety. Vasospasm and plaque rupture could not be excluded in this case, due to resolution of cardiac complications prior to establishing a seizure-free baseline. However, our patient met all the other criteria for SIC and resolution of cardiac injury occurred within four days, making an infarct unlikely. This patient never requires hemodynamic pressor augmentation, making cardiogenic shock very unlikely.

This raises the question of whether the current guidelines are adequate and necessary for diagnosing this condition. Another important question to consider is how to best delineate subtypes of stress-induced cardiomyopathy. Our patient exhibited no apical ballooning and other case series have shown patients to have either dyskinesia/akinesia in wall motion or mid-ventricular ballooning especially following convulsive seizures [5, 8, 9]. Likewise, the association with varying stressors may involve other pathophysiologic pathways other than catecholamine surge that are not fully understood. Of consideration is the possibility of a neurocardiac pathway, as similar cardiomyopathy findings have been seen in intracranial hemorrhage [3, 4]. Recent case studies have shown an increased risk of SIC after seizure without classic symptoms of acute coronary syndromes (chest discomfort, radiating pain, shortness of breath, or crushing chest pain) [6].

Recent case studies and case series have elucidated the presence of nonapical involved cardiomyopathy associated with various stress precursors [4, 6]. Current subtypes recognized are apical and nonapical regional wall dysfunction, which in some instances may be associated with a subset of precipitating stressors. Recent case studies have involved patients with nonapical ballooning cardiomyopathy following hemorrhagic stroke or seizures in particular [5, 9, 10]. The case presented here involves recurrence of nonapical stress-induced cardiomyopathy following status epilepticus. SIC is observed to have recurrence by up to 11.4% over the first 4 years [9]. Future case series should review the incidence of recurrence of SIC associated with seizures. Additional topics of interest, assessing for recurrence of SIC as associated with type and severity of epileptic activity, and consideration of antiepileptic therapy and serum concentrations prove to increase risk of SIC and recurrence.

The current diagnostic standard for patients suspicious for SIC requires ruling out acute myocardial infarction, acute coronary syndrome, myocarditis, neurogenic pulmonary edema, and nonischemic cardiomyopathy. Therefore, ECG, TTE, cardiac enzymes (especially troponins), and cardiac catheterization should occur in order to rule out other causes [3, 8]. Diagnostic criteria include the following: transient apical or mid-ventricular ballooning with subsequent left ventricle (LV) dyskinesia/akinesia that extends beyond one coronary distribution, the absence of CAD or acute plaque rupture by cardiac catheterization as the source of myocardial dysfunction, new ECG changes such as transient ST segment elevation or diffuse T-wave inversions, and a mild to moderate elevation of troponins. The underlying pathophysiology is believed to be related to catecholamine surge generating a transient stress-induced demand ischemia [3, 4, 8].
